# Pancytopenia as a Complication of Low-Dose Methotrexate in a Septuagenarian: A Rare Presentation

**DOI:** 10.7759/cureus.8492

**Published:** 2020-06-07

**Authors:** Tejaswi Kanderi, Janet Chan Gomez, Max M Puthenpura, Keerthi Yarlagadda, Mounika Gangireddy

**Affiliations:** 1 Internal Medicine, University of Pittsburgh Medical Center (UPMC) Pinnacle, Harrisburg, USA; 2 Internal Medicine, Drexel University College of Medicine, Philadelphia, USA

**Keywords:** pancytopenia, methotrexate, leucovorin, impaired renal function

## Abstract

Methotrexate (MTX) is an antimetabolite that was initially developed as a chemotherapeutic agent to treat malignancies but later used extensively to treat rheumatological conditions. MTX-induced toxicity is dose- and duration-dependent. Myelosuppression is a rare but fatal complication of MTX that can occur even with low doses used for inflammatory conditions. Multiple factors such as age, renal impairment, and nutritional status increase the risk of developing MTX toxicity. Frequent monitoring of symptoms and lab values are the hallmarks of prompt diagnosis and prevention of complications. Clinicians should have a high degree of suspicion to diagnose pancytopenia secondary to MTX especially in patients with multiple confounding comorbidities. We present the case of a 79-year-old male who presented with mucositis and pancytopenia diagnosed to be secondary to weekly MTX for giant cell arteritis leading to anemia and septic shock causing death.

## Introduction

Methotrexate (MTX) is an antimetabolite that was initially developed as a chemotherapeutic agent in the management of malignancy. It was only later that MTX at low doses gained popularity as a disease-modifying agent for autoimmune diseases. Giant cell arteritis (GCA) is a prolific autoimmune disorder defined as a systemic inflammatory disease of the medium and large arterial vessels, with high prevalence in the elderly population [1]. Active GCA has a high risk of permanent vision loss if left untreated [2]. Histological confirmation with temporal artery biopsy is the gold standard for diagnosis; however, sensitivity is low given the skip pattern of inflammation [1]. Glucocorticoids are the mainstay of treatment, but in cases of anticipated glucocorticoid side effects where patients have a protracted treatment course or exacerbating comorbid conditions such as diabetes mellitus, glucocorticoid-sparing agents are used as an adjunct therapy or instead of traditional steroid treatment [2,3]. MTX is a widely used alternative therapy due to its ability to dampen systemic auto-inflammatory response [3,4]. However, like most therapeutics, MTX has its side-effect profile, with one of the most drastic and complicated adverse outcomes being medication-induced pancytopenia. Careful monitoring and clinical discretion should be used in following patients with MTX-induced pancytopenia until a resolution, as the condition carries a mortality of around17-44% [5].

Here we discuss the case of a 79-year-old male who presented with pancytopenia attributed to MTX toxicity after excluding other possible etiologies.

## Case presentation

A 79-year-old Caucasian male with a medical history of anemia of chronic disease, chronic kidney disease (stage 3), iron deficiency anemia, insulin-dependent type 2 diabetes mellitus, hypertension, coronary artery disease, paroxysmal atrial fibrillation (Afib), GCA, IgG kappa MGUS (monoclonal gammopathy of undetermined significance), and internal hemorrhoids presented to the ED with complaints of generalized weakness and mouth sores for the past two weeks. Vitals on presentation include a temperature of 37.5 °C (99.5 °F), pulse rate of 79, respiratory rate of 19 breaths per minute, blood pressure of 176/66, and saturation level of 100% on room air. The physical examination was significant for large circular oral ulcers, with surrounding erythema bilaterally in the mouth and throat, and the rest of the physical examination including the neuroexamination was unremarkable. The patient has a 30 pack-year smoking history and drinks alcohol occasionally. 

Labs included hemoglobin of 7.9 g/dL (baseline: 10 g/dL), white blood cell count of 0.50 K/uL (baseline 9-10 K/uL), and platelets of 12 K/uL (baseline: 300-400 K/uL). White blood cell count with differential revealed neutrophils of 14% (reference: 50-70%) and absolute neutrophil count (ANC) of 70 cells/uL (reference: 1,800-7,400) with +2 anisocytosis, microcytosis, +2 poikilocytosis, +1 schistocytosis along with dhole bodies, atypical lymphocytes, and toxic granulation.

Labs were also significant for red cell distribution width (18% [reference: 12- 15%]), creatinine (2.57 mg/dL [baseline: 1.8-2.0 mg/dL]), glomerular filtration rate (23 mL/minute [baseline: 40-60 mL/minute]), aspartate transaminase (14 U/L [reference <40 U/L]), alanine transaminase (37 U/L [reference: <60 U/L]), alkaline phosphatase (117 U/L [reference: <100 U/L]), ferritin (>5,000 ng/mL [reference: <300 ng/mL]), iron (57 mcg/dL [reference: 60-150 mcg/dL]), iron saturation (17% [reference: 20-50%]), iron-binding capacity (150 mcg/dL [reference: 250-450 mcg/dL]), and sedimentation rate (110 mm/hour [reference: 0-20 mm/hour]); mean corpuscular volume and folic acid were normal. Peripheral smear showed rare megaloblasts with no blasts besides anemia, leucopenia, and thrombocytopenia.

Of note, his dabigatran (anticoagulation for Afib) was held for the past month due to recurrent episodes of epistaxis. The patient has been on PROCRIT® for anemia of chronic disease and has had intravenous iron in the past for iron deficiency anemia.

**Figure 1:**
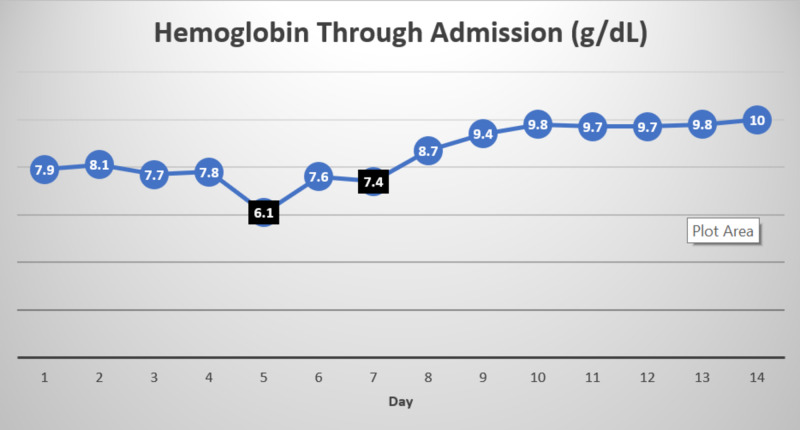
Trend in hemoglobin Day 1 is the day of admission. Values marked in black represent the values that prompted blood transfusion.

**Figure 2:**
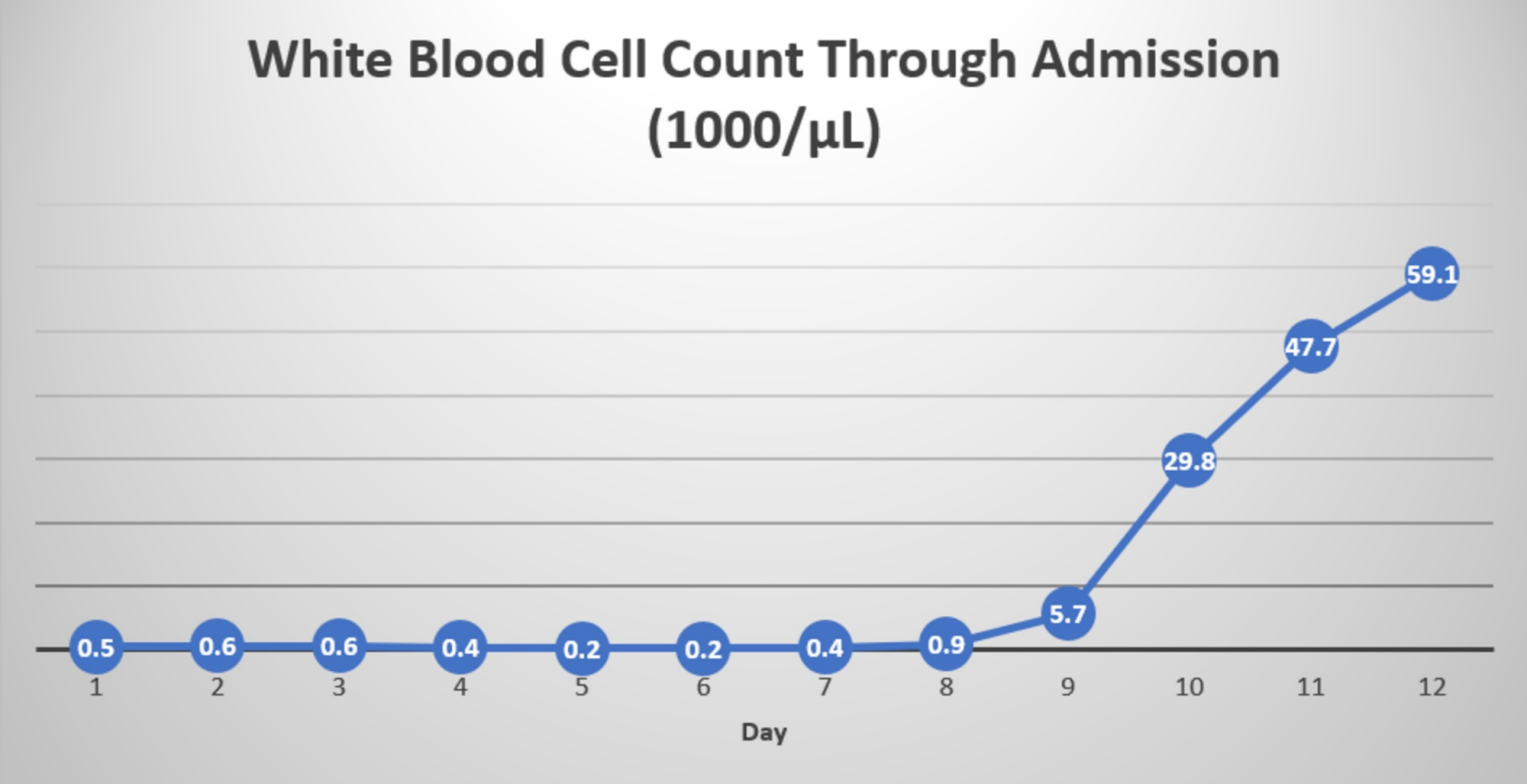
Trend in white blood cell count Day 1 is the day of admission.

**Figure 3:**
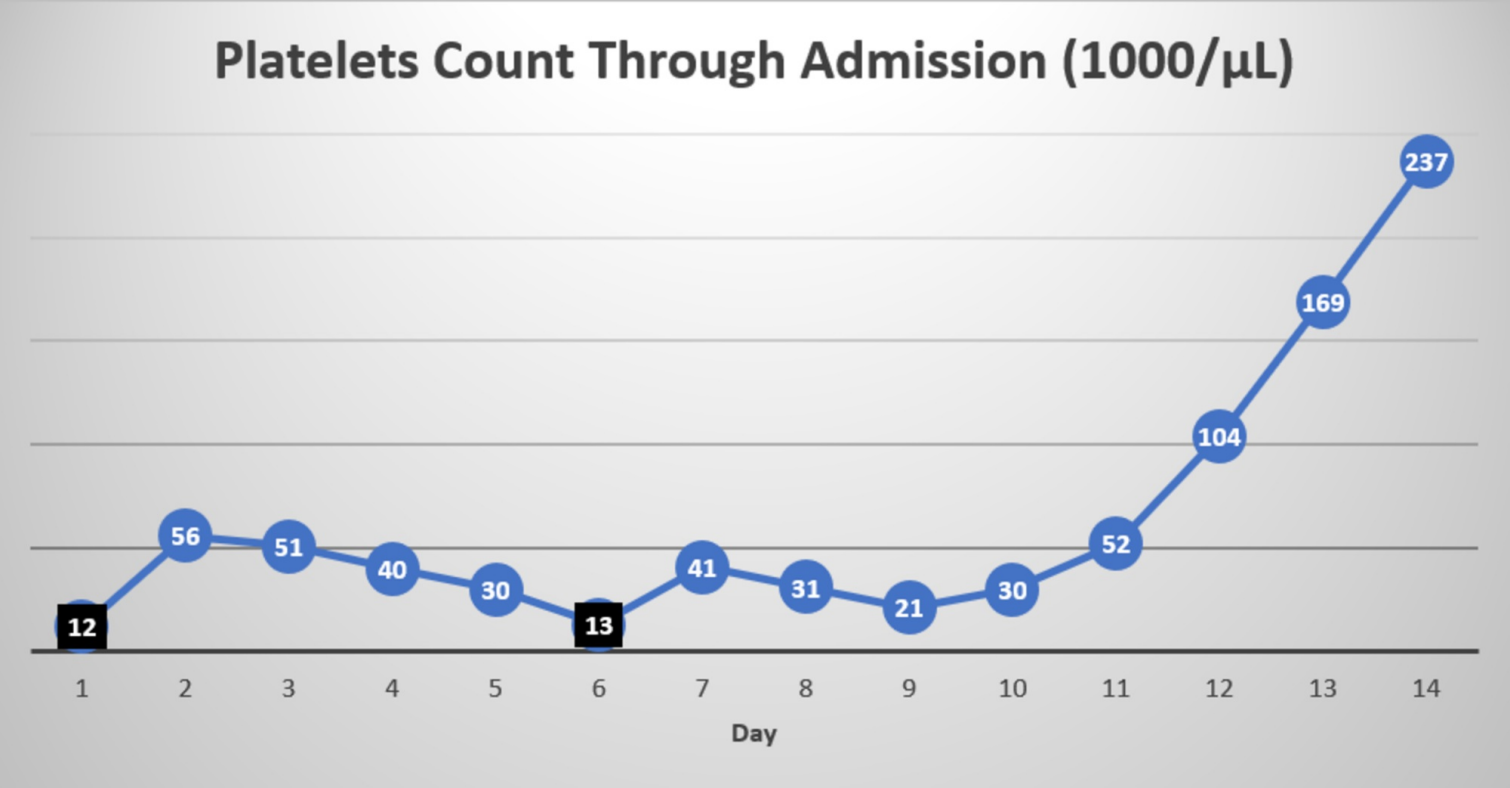
Trend in platelets Day 1 is the day of admission. Values marked in black represent the values that prompted platelet transfusion.

The patient was transfused one unit of platelets and was started on neutropenic precautions and broad-spectrum antibiotics with cefepime and vancomycin. He underwent esophagogastroduodenoscopy and colonoscopy, which revealed mild gastritis and internal hemorrhoids, respectively, with no active bleeding. Blood cultures grew methicillin-resistant coagulase-negative staphylococci (MRCNS) sensitive to vancomycin and pan-sensitive Escherichia coli. Careful medication reconciliation revealed that the patient was started on oral MTX 15 mg once every week, folic acid 1 mg daily, and prednisone 2 mg once daily less than six months ago for his GCA. Onset and worsening of pancytopenia and mucosal ulcers correlated to the period of starting the medications. The impaired renal function might have also contributed to MTX toxicity as the drug is excreted primarily by kidneys. MTX levels on admission were not available. The hematology team was consulted for assistance in the treatment of MTX-induced pancytopenia, resulting in discontinuation of MTX and starting intravenous leucovorin 10 mg/m^2^ every six hours for four doses along with folic acid on the day of admission. Throughout the hospital course, the patient also received blood and platelet transfusions with a hemoglobin goal of 7.5 to 8 g/dL and a platelet goal of 20 K/uL if no bleeding. Pancytopenia significantly improved over the next week with a white blood cell count of 5.7 k/uL, ANC of 730 cells/uL, hemoglobin of 9.7 g/dL, and platelets of 104 K/uL. Please refer to Figures 1-3 for trends in hemoglobin, white blood cell count, and platelets, respectively.

Oral mucositis gradually worsened to a point where the patient could tolerate any oral intake, leading to percutaneous endoscopic gastrostomy tube placement and discharge to long-term acute care (LTACH) facility given the overall deconditioning.

We did not see a need for bone marrow biopsy as the decrease in cell counts correlated with the start of MTX, and cell counts improved with discontinuation of MTX and treatment with folinic acid within one week. Interestingly, the patient underwent a bone marrow biopsy in 2009 and was diagnosed with MGUS with no evidence of lymphoproliferative process or clonal expansion of plasma cells; since then he was being followed by hematology and had normal paraproteins a year before the presentation.

He completed the antibiotic course as recommended by the infectious disease team. His course at LTACH was complicated by a v-fib arrest, return of spontaneous circulation (ROSC) was achieved, and the patient was transferred to the intensive care unit, where his clinical status further worsened despite being on multiple vasopressors, leading the family to change him to the DNR (do-not-resuscitate) status; shortly thereafter the patient succumbed to cardiac arrest (PEA [pulseless electrical activity] arrest).

## Discussion

MTX is a folate antagonist that inhibits dihydrofolate reductase (DHFR), preventing the conversion of dihydrofolate to tetrahydrofolate and thus blocking the synthesis of purines and pyrimidines and, therefore, inhibiting DNA, RNA, and protein synthesis. However, coming to its effect against autoimmune diseases, multiple mechanisms such as inhibition of T-cell activation, selective downregulation of B cells, and inhibition of methyltransferase activity are thought to play a role in addition to inhibiting DHFR [6,7].

MTX-induced pancytopenia is dose- and duration-dependent and is seen in approximately 1.4% of the reported side effects, with a female preponderance (62.51%), with around 59% in patients above 60 years of age [8,9]. The effects of pancytopenia include anemia, leukopenia, and thrombocytopenia. This results in systemic manifestations including fatigue due to low hemoglobin, infections secondary to leukopenia and/or neutropenia, bleeding, and ecchymosis from decreased platelet count. Monitoring patients for clinical signs and symptoms of pancytopenia is crucial in timing for tapering or discontinuing treatment and administering rescue therapy such as leucovorin (folinic acid), which has demonstrated some efficacy in expediting recovery [10].

MTX toxicity is reported to start with stomatitis and progress to pancytopenia, which can occur at any time of the treatment [11]. Myelosuppression/ pancytopenia as a complication of MTX is well known, but low doses causing the same is not well documented, which needs strong emphasis as the drug has gained wide acceptance among rheumatologists due to its efficacy and relatively safe therapeutic window in a wide variety of inflammatory rheumatologic conditions including GCA [12,13]. Case reports and meta-analysis report a decrease in the risk of relapse and limiting the duration of steroids by using 10-15 mg/week of MTX as adjunctive therapy in patients with GCA [14,15].

Possible risk factors include age and age-related decline in renal function owing to increased levels of MTX. Hypoalbuminemia also increases the risk of toxicity due to increased levels of free MTX than albumin-bound MTX [16]. Studies also reported nutritional status, polypharmacy, use of other antifolate drugs, and prescribing errors as other risk factors [11]. Poor nutritional status has been reported to be a risk factor due to prolonged half-life and a significant decrease in clearance in undernourished patients [16,17]. Mucositis and ulceration, in turn, lead to poor nutritional intake and worsening of renal function, and also serve as sites of serious infection. In our patient age, a poor nutritional status due to mucositis and acute-on-chronic decrement of renal function may have also contributed to the toxicity.

The American College of Rheumatology recommends complete blood count (CBC), serum creatinine, and transaminase tests to be performed at baseline before initiating MTX therapy, with monitoring every 2-4 weeks for the first three months, 8-12 weeks for the next three months, and once every 12 weeks thereafter [18]. Recovery time from pancytopenia varies depending on other comorbidities and concomitant infection; however, studies reported median recovery time around four to six days [11].

MTX-induced pancytopenia is commonly reported with the treatment of rheumatoid arthritis (RA) than with GCA. RA by itself can cause anemia, neutropenia, and thrombocytopenia (in case of Felty syndrome with splenomegaly), whereas GCA can cause anemia, although other hematological manifestations are rarely reported, drawing a possibility of disease process augmenting pancytopenia in addition to MTX in RA.

Folic acid supplementation may reduce MTX toxicity. Studies also report that folic acid can prevent MTX-induced hyperhomocysteinemia, thus ensuring cardioprotection [19]. However, MTX toxicity has been reported even with supplementation of folic acid, just like in our patient. Leucovorin is thought to prevent stomatitis and also can aid in quick recovery from toxic effects. Studies also report the use of G-CSF (granulocyte colony-stimulating factor and steroids for MTX-induced myelosuppression in patients with RA, but there is a need for further studies to confirm similar findings in GCA [20].

Frequent lab monitoring and careful increments of dose, if needed, are the cornerstone in preventing and prompt diagnosis of MTX toxicity. We also think that MTX-induced pancytopenia is underreported and overlooked especially in patients with multiple comorbidities with the possibility of misattributing pancytopenia to other conditions.

## Conclusions

Caution should be taken before prescribing MTX to the elderly, especially those with impaired renal function. In addition to periodic monitoring with CBC, patients and their families should be educated about the possible toxic effects and symptoms such as stomatitis, fatigue, and fever, as long-term use of MTX for inflammatory conditions is becoming prevalent. Clinicians should have a high degree of suspicion to diagnose MTX-induced pancytopenia for the appropriate management, thereby avoiding fatal outcomes.
